# Automated DNA extraction platforms offer solutions to challenges of assessing microbial biofouling in oil production facilities

**DOI:** 10.1186/2191-0855-2-60

**Published:** 2012-11-20

**Authors:** Athenia L Oldham, Heather S Drilling, Blake W Stamps, Bradley S Stevenson, Kathleen E Duncan

**Affiliations:** 1 The Department of Microbiology and Plant Biology, University of Oklahoma, 770 Van Vleet Oval GLCH #136, Norman, OK 73019, USA; 2 University of Oklahoma Biocorrosion Center, Norman, OK 73019, USA; 3 The Institute for Energy and the Environment, University of Oklahoma, 100 E. Boyd Street, Norman, OK, 73019, USA

**Keywords:** 16S rRNA gene, Automated nucleic acid extraction platforms, Microbial biofouling, Bacterial community, Oilfield microbiology

## Abstract

The analysis of microbial assemblages in industrial, marine, and medical systems can inform decisions regarding quality control or mitigation. Modern molecular approaches to detect, characterize, and quantify microorganisms provide rapid and thorough measures unbiased by the need for cultivation. The requirement of timely extraction of high quality nucleic acids for molecular analysis is faced with specific challenges when used to study the influence of microorganisms on oil production. Production facilities are often ill equipped for nucleic acid extraction techniques, making the preservation and transportation of samples off-site a priority. As a potential solution, the possibility of extracting nucleic acids on-site using automated platforms was tested. The performance of two such platforms, the Fujifilm QuickGene-Mini80™ and Promega Maxwell®16 was compared to a widely used manual extraction kit, MOBIO PowerBiofilm™ DNA Isolation Kit, in terms of ease of operation, DNA quality, and microbial community composition. Three pipeline biofilm samples were chosen for these comparisons; two contained crude oil and corrosion products and the third transported seawater. Overall, the two more automated extraction platforms produced higher DNA yields than the manual approach. DNA quality was evaluated for amplification by quantitative PCR (qPCR) and end-point PCR to generate 454 pyrosequencing libraries for 16S rRNA microbial community analysis. Microbial community structure, as assessed by DGGE analysis and pyrosequencing, was comparable among the three extraction methods. Therefore, the use of automated extraction platforms should enhance the feasibility of rapidly evaluating microbial biofouling at remote locations or those with limited resources.

## Introduction

Microbial biofouling is a significant problem facing many different systems including industrial (e.g. fuel production, food production, drinking-water, etc.), marine (e.g. ship ballast tanks) and medical (e.g. catheters) (Bixler and Bhushan 2012). Biofilm formation, or the accumulation of water-borne microorganisms and their associated extrapolymeric substances (EPS) on wetted surfaces, is a major contributor to biofouling and becomes an economic liability when it exceeds some threshold of interference, resulting in material damage, production loss, or elevated health risks (Murthy and Venkatesan 2009). Therefore, rapid sample processing and analysis is necessary for prompt microbial biofouling assessment. Due to limited space, resources, or expertise, samples from the facilities at risk are often shipped to research or commercial laboratories for nucleic acid extraction and analysis, where the efficacy of antifouling approaches, such as biocide treatment or physical biofilm removal (Quarini and Shire 2007), can be rapidly deduced using molecular-based approaches. These approaches include amplification of both the 16S rRNA gene and functional genes to identify specific microbes and qPCR to quantify target genes (Smith and Osborn 2009). The caveats of shipping samples for extraction in lieu of extracting on-site include: 1) shipping materials that could be considered "hazardous," and 2) the microbial community structure of the sample could shift during the time in transit from facility to lab, leading to erroneous results. Therefore, the ability to extract nucleic acids on-site and within hours of sampling could bypass these two caveats and hasten microbial biofouling assessment and treatment.

Successful molecular-based studies rely on extraction of high-quality nucleic acids from complex samples (i.e. production waters, biofilm material, sediment, etc.). These types of samples can contain factors that interfere with cell lysis, nucleic acid capture, or polymerase activity (Wilson 1997). Therefore, improvements in extraction performance, such as increased lysis or inhibitor-removal technologies increase the likelihood of obtaining nucleic acids for use in downstream PCR-based applications (van Der Kraan et al. 2011). All nucleic acid extraction protocols require the same basic steps: cell lysis to release nucleic acids from microbes, removal of inhibitors that can interfere with downstream applications, and nucleic acid mobilization, purification, and elution into a suitable buffer. The ease of use and time requirements, however, will vary based on the degree of automation and requirements for additional equipment (Table 1). Due to the complexity of industrial and environmental sample types, commercially available kits are recommended for distinct sample types. While kits have made extracting DNA and RNA from complex samples feasible in a laboratory setting, the manual steps and additional equipment requirements which often include an incubator, microcentrifuge, and physical lysis equipment make preparation difficult for personnel with limited training, few laboratory resources, or in remote locations.

**Table 1:** Comparison of DNA extraction platforms

**Platform**	**PowerBiofilm**	**QuickGene**	**Maxwell**
Ease of operation	Manual	Semi-automated	Automated
**Dedicated instrument**	No	QuickGene-Mini80	Maxwell 16
Cost^a^		$1500	$25000
**Processing steps**			
Sample preparation	Manual	Manual	Manual
Cell lysis	Manual	Manual	Automated
Inhibitor removal	Manual	Manual	Automated
Nucleic acid mobilization	Manual	Automated	Automated
3 x Washes	Manual	Automated	Automated
Elution	Manual	Automated	Automated
Total time	120 min (n = 10)	60 min (n = 8)	45 min (n = 16)
**Additional equipment**			
Microcentrifuge	Yes	Yes	No
Incubator (>55°C)	Yes	Yes	No
Physical lysis equipment^b^	Yes	Yes	No
Refrigerator (4°C)	Yes	No	No
**Consumable supplies**			
Cost (per sample)	$6.50	$2.60	$6.33

^a^Approximate list price at time of purchase.

^b^Physical lysis equipment: a bead-beater for PowerBiofilm and a rotisserie for Quickgene.

Our lab has extensive experience with traditional phenol-chloroform DNA extractions and with using a broad array of commercially available extraction kits, both of which require ancillary laboratory equipment. The goal of this study was to test the feasibility of using more automated extraction approaches on biofilm material scraped from oilfield pipelines, as an example of the types of complex samples encountered in industrial situations. We hypothesized that the two automated platforms chosen would perform equivalently to a widely used manual extraction kit when compared by a set of standard PCR-based analyses. The rationale for this study was to determine if more automated extraction platforms would enable personnel, untrained in molecular biology or with limited laboratory resources, to extract DNA on-site and within hours to preserve sample integrity. The two automated extraction platforms tested were the semi-automated QuickGene-Mini80™ (Autogen/FujiFilm, Holliston, MA) and the automated Maxwell®16 (Promega, Madison, WI) platforms. Both systems were designed to extract nucleic acids from various tissues and cell types in clinical labs (Affolabi et al. 2012) and both systems have proven successful for a wider variety of additional sample types including spores (Shipley et al. 2012), plant leaves, seeds, and fungi (Affolabi et al. 2012; Foley et al. 2011; Khokhar et al. 2011; Schagat et al. 2007). The manual kit, PowerBiofilm DNA Isolation Kit (MOBIO Laboratories, Carlsbad, CA), was designed to extract DNA from biofilm material and is representative of the kits widely used by environmental microbiologists (Ferrando and Tarlera 2009; McBeth et al. 2010). For the three test samples, we compared extraction platform ease of use and DNA yields. A series of PCR-based analyses was then used to assess DNA extract quality and effect on microbial community profiles.

## Materials and methods

### Pipeline biofilm samples

Three samples scraped from the inner surface of oilfield pipelines (i.e. pigged pipeline material) were collected and stored at −80°C. Two of the samples, designated "A" and "B", originated from pipelines carrying produced water being returned to the formation to maintain pressure. A third sample, "C", originated from a pipeline as part of a seawater injection system for secondary oil recovery. Samples A and B contained crude oil, corrosion products such as iron sulfides and biofilm material (e.g. EPS). Sample C did not contain crude oil but did contain lesser quantities of corrosion products and biofilm material. For samples A, B, and C, DNA was extracted from ten aliquots (subsamples) for each extraction platform. For samples A and B (40 ml), each was thawed at 4°C, mixed, and ten replicate subsamples (0.5 ml) were dispensed into 2 ml conical screw-top tubes. Sample C (40 ml) was thawed, mixed, and ten replicate subsamples (1 ml) were dispensed into 2 ml conical tubes and concentrated by centrifugation for 5 min at 14000 × *g,* removing 0.5 ml of the supernatant and re-suspending the remaining volume. This concentration of biomass was deemed necessary for sample C, as initial studies revealed it contained 1/10^th^ of the biomass of samples A or B (personal communication, Kathleen Duncan).

### MOBIO PowerBiofilm extraction platform

The PowerBiofilm DNA Isolation Kit (MOBIO Laboratories) was used to manually extract DNA from ten replicate subsamples of samples A, B, and C according to the manufacturer's instructions. Specifically, the contents of a PowerBiofilm bead tube, and 350 μl of buffer BF1 and 100 μl of buffer BF2 were added to each sample tube. Samples were vortexed and incubated at 65°C for 5 min. Physical lysis of cell material was accomplished using the Mini-BeadBeater-16 (BioSpec Products, Bartlesville, OK) at 3450 oscillations/min for 2 min. Samples were spun at 13000 × *g* for 1 min. Supernatants were transferred to fresh tubes and 200 μl of buffer BF3 were added; samples were incubated at 4°C for 5 min and subsequently spun for 1 min. Supernatants were transferred to fresh tubes and 900 μl of buffer BF4 were added and samples mixed. Samples were loaded onto a PowerBiofilm spin filter column and spun for 1 min repeatedly until all sample was collected onto the filter. Filters were washed with 650 μl of buffer BF5 followed by buffer BF6 and ended with a final spin for 2 min. DNA was eluted in 100 μl of buffer BF7 with a final spin for 1 min.

### Fujifilm QuickGene-Mini80 extraction platform

DNA was extracted from ten replicate subsamples of A, B, and C using the QuickGene DNA Tissue Kit S with the semi-automated QuickGene-Mini80 instrument (Autogen/FujiFilm, Holliston, MA) following manufacturer's instructions. Cell lysis was facilitated by adding 180 μl of Tissue lysis buffer (Autogen/FujiFilm) and 20 μl Proteinase K to each sample tube and mixing with a Thermolyne LabQuake Rotisserie Tube Shaker (ThermoScientific/Barnstead, Waltham, MA) for 30 min at 55°C. Samples were spun at 10000 × *g* for 3 min. The supernatants were transferred to a new tube and 20 μl RNase A were added and incubated for 2 min. Next, 180 μl Lysis buffer and 240 μl ethanol (>99%) were added and the sample was vortexed for 15 s. Samples were transferred to QuickGene cartridges and placed within the QuickGene Mini80 apparatus, and DNA binding, washing, and elution were accomplished through pressurization. DNA was eluted with 200 μl Elution buffer.

### Promega Maxwell 16 extraction platform

DNA was extracted from ten replicate subsamples of A, B, and C using the automated Maxwell 16 Cell Total RNA Purification Kit with the Maxwell 16 Instrument (Promega) set to the LEV (low elution volume) configuration. Specifically, samples were loaded into the pre-dispensed reagent cartridges along with 400 μl RNA lysis buffer and 400 μl RNA dilution buffer. Elution tubes containing 100 μl nuclease-free water, plungers, and cartridges containing the sample and buffers were placed within the instrument and all subsequent steps were automated following the pre-programmed DNA extraction protocol. The DNA-removal steps of the Total RNA Purification Kit protocol were omitted to preserve the DNA (Promega Field Application Specialist, personal communication).

### Evaluation of extracted DNA yield

DNA extracts from the subsamples were analyzed by gel electrophoresis and quantified using fluorometry to compare the reproducibility of extraction among replicate samples. To visualize the DNA fragment, 10 μl of each extract was analyzed alongside 2 μl of Lambda DNA/EcoRI+HindIII marker (ThermoFisherScientific/Fermentas, GlenBurnie, MD) on a 1% agarose gel (wt/vol) stained with SYBR®Safe (Invitrogen, Carlsbad, CA). Gels were visualized and the image captured using the Gel Logic 112 Imaging System and Molecular Imaging Software v5 (Carestream, WoodBridge, CT). DNA extracts were quantified using the Qubit 2.0 fluorometer with the dsDNA or RNA reagents according to the manufacturer’s protocol (Invitrogen/Life Technologies, Carlsbad, CA). GraphPad Prism5 (GraphPad Software, San Diego, CA) was used to generate box-and-whisker plots to visualize the degree of variation in DNA yields among replicate extractions. Upper and lower whiskers illustrate the maximum and minimum DNA yields, respectively, and the median DNA yield separates the box into upper (75%) and lower (25%) quartiles.

For each of the three platforms, the ten subsample DNA extracts were pooled to generate DNA stocks for subsequent analyses to assess DNA extract quality and its effect on the microbial community structure, while minimizing the effect of small sample volumes. For each of the three samples A, B, and C, the total amount of DNA extracted from equivalent sample volumes for each platform was determined by multiplying the concentration of each of the ten subsample extractions by its elution volume and summing the products.

### Evaluation of extracted DNA quality using qPCR analysis

DNA extraction quality was evaluated for PCR inhibition via amplification of the bacterial 16S rRNA gene in undiluted versus diluted DNA extracts. Briefly, 30 μl reactions contained 15 μl 2 × SYBR®Green PCR Master Mix (Life Technologies, Carlsbad, CA), 0.5 M betaine (N,N,N-Trimethylglycine) (Sigma-Aldrich, St Louis, MO), 250 nM of the primer 27f (5′-AGAGTTTGATCCTGGCTCAG) and 125 nM of the primer 338r (5′- TGCTGCCTCCCGTAGGAGT) as described in Stevenson et al. ( 2011). Thermal cycling, data acquisition and analyses were carried out with the StepOnePlus™ Real-Time PCR System and StepOne Software v2.1 (Life Technologies). Cycling conditions were: 95°C for 10 min followed by 40 cycles of 95°C for 30 s, 55°C for 45 s, 72°C for 45 s, and ended with a melt curve stage of 95°C for 15 s, 60°C for 1 min, and 95°C for 15 s. Image capture was at 72°C. DNA was assayed in triplicate at undiluted, 1:10, and 1:100 dilutions. A 10-fold dilution series of a control plasmid was assayed in duplicate and spanned 10^3^ to 10^9^ copies. Molar concentrations were converted into 16S copies based on the following assumptions: the average molecular mass of a double strand DNA base pair (bp) is 6.6 × 10^11^ ng mol^-1^, Avogadro’s number of copies mol^-1^ is 6.02 × 10^23^ (Mc Kew and Smith 2010):

$$\text{Copies =}\;\frac{\text{concentration (ng per μl) ×}\;6{\text{.02 × }10}^{23}\left(\text{copies per mol}\right)}{\text{length (bp) ×}\;6{\text{.6 × }10}^{11}\;\text{(ng per mol})}$$

### Evaluation of the influence of DNA extraction platform on microbial community composition using denaturing gradient gel electrophoresis (DGGE)

A DGGE analysis of amplified bacterial 16S rRNA genes was used to evaluate potential biases in cell lysis between extraction platforms. Briefly, 2 μl of DNA were amplified by end-point PCR in 25 μl reactions. Each reaction contained: 0.625 U DreamTaq™ polymerase (Fermentas, Glen Burnie, MD), 0.2 mM dNTP mixture, 0.5 M betaine, 1 × DreamTaq Buffer (Fermentas), and 100 nM each of forward primer GM5F (5′-CCTACGGGAGGCAGCAG) containing the GC clamp on the 5'-end and reverse primer D907R (5′-CCCCGTCAATTCCTTTGAGTTT) (Santegoeds et al. 1999). Thermal cycling was carried out with a TC-512 thermal cycler (Techne, Burlington, NJ) using a touchdown PCR program from 65°C to 55°C. Conditions were 94°C for 4 min, followed by 2 cycles each of 94°C for 1 min, N°C for 1 min, and 72°C for 1 min, where N°C dropped 1°C from 65°C to 55°C, followed by 15 cycles at the 55°C annealing temperature and a final extension at 72°C for 10 min. For bacterial community analysis, 15 μl of each reaction was resolved on a 6% polyacrylamide, urea and formamide 40-60% denaturing gradient gel (100% denaturant = 7 M Urea and 40% formamide) (Muyzer et al. 1993) and run at 65 V for 16 h at 60°C. The gel was stained for 15 min in SYBR Safe (at 25 μl per 250 ml) and visualized as described above.

### Identification of microbial community composition among DNA extracts using high throughput sequencing of 16S rRNA gene libraries

To identify major bacterial taxa in samples A, B, and C DNA extracts, bacterial 16S rRNA gene libraries were generated from each extraction method modeled after the approach used by Wawrik et al. ( 2011). For each sample, triplicate 50 μl reactions contained 5 μl to 10 μl DNA, 1.25 U DreamTaq polymerase, 0.2 mM dNTP mixture, 0.5 M betaine, 1xDreamTaq Buffer (Fermentas), 250 nM 27f and 125nM 338r primers. Thermal cycling was carried out on a TC-512 thermal cycler (Techne) with the following conditions: 96°C for 3 min; 30 cycles of 96°C for 30 s, 55°C for 45 s, 72°C for 45 s; and a final extension at 72°C for 10 min. Triplicate reactions were pooled and purified using the Wizard PCR Preps DNA Purification System (Promega). From each of the purified PCRs, 5 μl was added to a second PCR containing barcoded PCR primers TiA-8nt-CA-27f (5′-CCATCTCATCCCTGCGTGTCTCCGACTCAGxxxxxxxxCAAGAGTTTGATCCTGG CTCAG) and TiB-CA-338r (5′-CCTATCCCCTGTGTGCCTTGGCAGTCTCAGCA TGCTGCCTCCCGTAGGAGT) for multiplexed pyrosequencing as described by Hamady et al. ( 2008). Each sample received a different tagged forward primer, containing a specific 8 nt ‘barcode’ sequence (designated by x), and samples were ‘tagged’ by re-amplification for six cycles. Barcodes are listed in Additional file 1: Table S1. The efficacy of the tagging reaction was confirmed by gel electrophoresis. Tagged PCR products were pooled in equimolar amounts and sequenced on a GS-FLX sequencer using the Titanium chemistry at (University of Oklahoma's Advanced Center for Genome Technology 2012).

The bacterial 16S rRNA gene libraries were analyzed using the bioinformatics software package, mothur ver1.24 (Schloss et al. 2009). An implementation of the Amplicon Noise algorithm was used to reduce the sequencing error incurred with pyrosequencing (Quince et al. 2011). Sequences were binned by barcode and screened to remove those containing errors in the forward primer or barcode. Unique sequences were trimmed to overlap a minimum of 200 base pairs and aligned against the SILVA reference alignment database (Pruesse et al. 2007) using the NAST-aligner (DeSantis et al. 2006). Sequences were pre-clustered using a single linkage algorithm (Huse et al. 2010) to reduce the number of spurious operational taxonomic units (OTUs) that would result from pyrosequencing errors, and subsequently screened for chimeras using UChime (Edgar et al. 2011). A distance matrix was generated and used to cluster sequences into OTUs at a 97% similarity level using the furthest neighbor algorithm. A representative sequence from each OTU was assigned a taxonomic classification based on the Ribosomal Database Project's naïve Bayesian classifier (Wang et al. 2007) at an 80% confidence threshold; and all richness and diversity measurements were calculated using the mothur software package based on a random subsampling subset of 1958 sequences to equal the number of reads in the smallest library. Using the generated distance matrix, an analysis of molecular variance (AMOVA) was used to determine if the observed differences in microbial diversity between sample groups or extraction methods was significantly different (Schloss 2008; Schloss and Handelsman 2008). Sequences were deposited in the short read archive of GenBank [GenBank: SRA052225].

## Results

### DNA extraction platform ease of use and cost considerations

The ease of use and cost parameters for the three extraction platforms are compared in Table 1. For PowerBiofilm, all steps from sample preparation to DNA elution were manual and additional equipment for sample processing included a microcentrifuge, bead-beater, incubator, and refrigerator. With multiple centrifugations, sample transfers, and incubation steps, the total processing time was approximately 120 min for ten samples. For the QuickGene Mini-80, initial sample processing steps were similar to that of PowerBiofilm with centrifugations and incubations for sample preparation and cell lysis, but DNA binding, washes, and elution were streamlined to process eight samples in parallel using pressure filtration technology. The total processing time was approximately 60 min, which included a 30 min incubation step for cell lysis. The Maxwell 16 platform required the least manual manipulation, with the transfer of sample plus lysis and dilution buffers to pre-filled reagent cartridges. The only other manual steps required for extraction set up were the addition of elution buffer, collection tubes and plungers into the cartridge-holder. All subsequent steps from cell lysis to nucleic acid elution were fully automated using pre-programmed settings and up to 16 samples could be processed in parallel. Extraction times for the two more automated platforms were less than half that as the manual method. Additional equipment requirements for sample processing were similar for PowerBiofilm and QuickGene; both required a microcentrifuge and incubator for processing steps as well as equipment for cell lysis. No additional equipment was necessary for the Maxwell 16 platform aside from the instrument itself. With regard to price per sample, consumable supplies for PowerBiofilm and Maxwell were similarly priced, whereas QuickGene was approximately one-third less (Table 1).

### Comparison of DNA yield between extraction platforms from equivalent starting sample volumes

**Figure 1:**
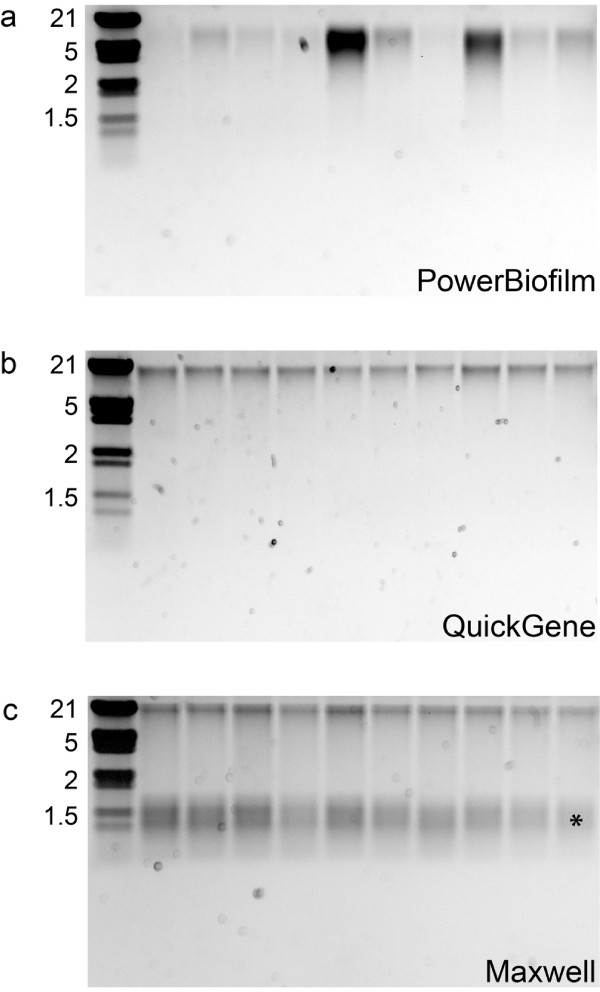
**Agarose gel analysis of sample A DNA extracts.** Comparison of DNA fragment size and relative quantity among subsamples. An aliquot (10 μl) of DNA extracted from replicate subsamples of sample A using the a) PowerBiofilm, b) QuickGene and c) Maxwell methods. Low weight nucleic acid (RNA) species in Maxwell extractions are indicated by an asterisk. Sizes (kb) of bands in the Lambda DNA/EcoRI+HindIII marker (left lane) are indicated.

Biofilm material scraped from the inner surface of three separate oil pipelines were initially extracted as ten subsamples to compare extraction reproducibility among replicate samples. The DNA fragment was visualized by gel electrophoresis and the gel images for sample A extracts are shown for a visual comparison between the three platforms (Figure 1). The PowerBiofilm method (P) extracted appreciable amounts of DNA from the ten subsamples, but DNA yields varied widely among them (Figure 1a). This result indicated a low consistency in extraction among replicate samples. By contrast, the QuickGene platform demonstrated more uniformity among subsamples (Figure 1, compare panel a to b), as did the Maxwell (Figure 1, compare panel a to c). RNA was also extracted using the Maxwell system, and was visible as a low molecular weight band (Figure 1c).

**Figure 2:**
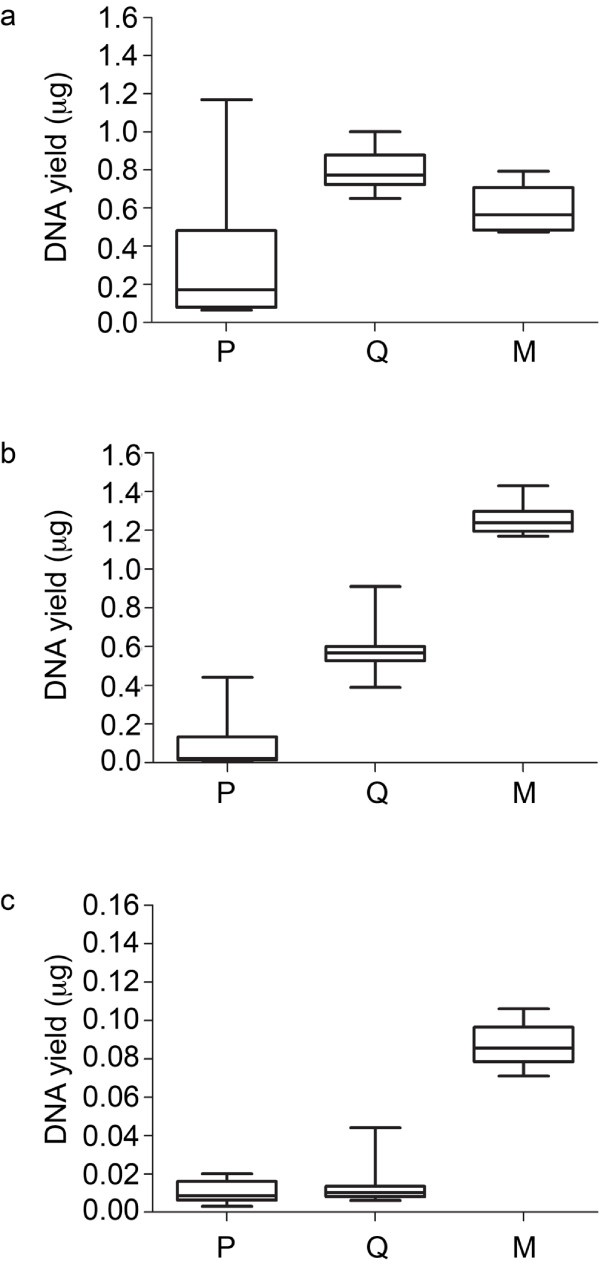
**Illustration of DNA extraction variability among replicate subsamples.** Box and whisker plots of replicate extractions (n = 10) for **a**) sample A, **b**) sample B, and **c**) sample C using PowerBiofilm (P), QuickGene (Q) and Maxwell (M) methods. The lower and upper whiskers illustrate minimum and maximum yields, respectively and the median yield separates the box into upper and lower quartiles.

DNA yields were estimated for each extraction using Qubit fluorometry and box-plots were used to illustrate the level of variability in DNA yields among the ten replicate DNA extracts (Figure 2). For sample A PowerBiofilm extracts, DNA yields ranged more than 10-fold from 0.06 μg to 1.17 μg, with the median yield at 0.17 μg (Figure 2a). For QuickGene and Maxwell extracts, median values were higher than PowerBiofilm and DNA yields were more consistent among the replicates. For samples B and C, distances between upper and lower whiskers (i.e. maximum and minimum yields, respectively) were closest among replicates for the Maxwell and PowerBiofilm extracts, respectively (Figures 2b and 2c). Subsequently, the ten replicate DNA extracts from each platform were pooled to compare the total DNA yields from equivalent starting sample volumes. The total amount of DNA extracted from 5 ml of sample A was approximately 3.37 μg using PowerBiofilm. Yields were higher for QuickGene and Maxwell at 8.01 μg and 6.01 μg, respectively. For sample B, the automated platforms also increased DNA yields from 0.94 μg (PowerBiofilm) to 12.56 μg and 5.80 μg for Maxwell and QuickGene, respectively. Next, DNA yields from the lower biomass sample C extractions were compared. From 10 ml of sample C, DNA yields were comparable for PowerBiofilm and QuickGene at 100 ng and 130 ng, respectively, however DNA yield was increased by almost ten-fold (870 ng) using the Maxwell platform. Together, these data demonstrated that the Maxwell platform could increase the DNA yields from both the high- and lower-biomass samples. QuickGene could also increase yields from samples A and B but had a negligible effect on the low-biomass sample C. Together, these results demonstrated that more automated platforms extracted higher DNA yields than the manual approach from equivalent starting sample volumes.

### Assessment of extracted DNA quality (i.e. PCR inhibition) by qPCR amplification in undiluted and diluted DNA extracts

DNA extract quality was evaluated for PCR inhibition via amplification of the V1-V2 region of the bacterial 16S rRNA gene in undiluted versus diluted (1:10 and 1:100) DNA extracts (Table 2). The rationale being that gene copy estimates per ml original sample would be higher using the diluted versus undiluted DNA extracts, as potential PCR inhibitors would be diluted out (Stults 2001). Estimates of gene copies per ml original sample for sample A were ~10^9^ copies per ml, with PowerBiofilm and QuickGene setting the lower and upper limits, respectively. For sample B, PowerBiofilm estimates were 10^8^ copies per ml, whereas the two more automated extractions both showed ~10^9^ copies per ml. For sample C, estimated numbers of gene copies per ml increased from ~10^6^ for PowerBiofilm and QuickGene to ~10^8^ for Maxwell. While there was some variation between estimates for a given sample among dilutions, 16S rRNA gene estimates mirrored the DNA quantification data with higher estimates from those with higher DNA yields. Importantly, gene estimates were not higher in the undiluted versus diluted DNA samples suggesting that PCR inhibitors were effectively removed by all three extraction platforms and did not interfere with amplification of the bacterial 16S rRNA gene in the undiluted DNA extracts.

**Table 2:** **Evaluation of PCR inhibition via bacterial 16S rRNA gene amplification in undiluted (1x) versus diluted DNA extracts**^**a**^

	**1x**^**b**^	**1:10x**^**b**^	**1:100x**^**c**^
**Sample A**			
**P**	2.03±0.15x10^9^	2.07±0.02x10^9^	1.32±0.07x10^9^
**Q**	6.26±0.57x10^9^	6.06±0.13x10^9^	2.88±0.04x10^9^
**M**	2.55±0.26x10^9^	2.38±0.10x10^9^	2.46±0.08x10^9^
**Sample B**			
**P**	8.44±0.17x10^8^	7.33±0.10x10^8^	5.79±0.10x10^8^
**Q**	6.34±0.56x10^9^	6.32±0.13x10^9^	3.72±0.19x10^9^
**M**	4.70±0.08x10^9^	5.16±0.20x10^9^	3.95±0.23x10^9^
**Sample C**			
**P**	6.28±0.43x10^6^	6.83±0.85x10^6^	7.48±0.52x10^6^
**Q**	9.26±5.48x10^6^	9.59±0.28x10^6^	8.83±0.18x10^6^
**M**	1.74±0.04x10^8^	1.14±0.05x10^8^	1.15±0.01x10^8^

^a^Data shown are mean values (n = 3) and standard deviations of 16S rRNA gene copies per ml original sample.

^b^Standard curve: m = −3.67; y-int = 41.61; R^2^ = 0.99; Eff = 87.1%.

^c^Standard curve: m = −3.59; y-int = 41.15; R^2^ = 0.99; Eff = 89.7%.

### Effect of DNA extraction platform on bacterial community composition by DGGE analysis

**Figure 3:**
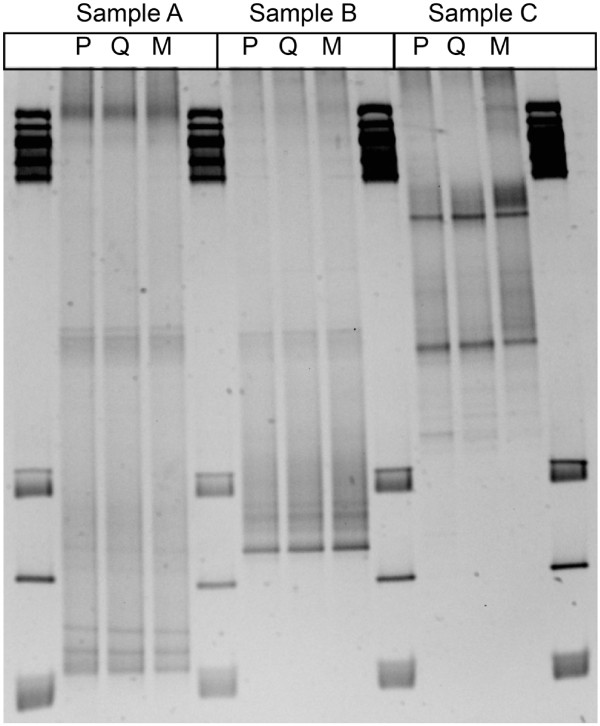
**DGGE analysis of bacterial 16S rRNA genes.** DNA extracts from samples A, B and C extracted using the PowerBiofilm (P), QuickGene (Q) and Maxwell (M) were used as template for DGGE. Gene ruler 1 kb plus ladder (3 μl) was run in lanes 1, 5, 9, and 13.

DNA extracts were further evaluated by amplification of the V3-V4 region of the bacterial 16R rRNA gene using end-point PCR. A DGGE analysis of these PCR products was evaluated to ask if the extraction platform influenced the bacterial community profile, as extraction method bias is well documented in the literature for difficult sample types, such as those containing gram-positive bacteria (Frostegard et al. 1999; Stach et al. 2001). While some variation in band intensity was observed, the overall banding patterns were similar among the three extraction methods for the same sample (Figure 3). These results suggest that the more automated extraction platforms lysed a greater proportion of cells from equivalent sample volumes rather than extracting DNA from group(s) of bacteria that were not lysed using PowerBiofilm.

### Identification of bacterial communities in DNA extracts using 454 pyrosequencing

**Figure 4:**
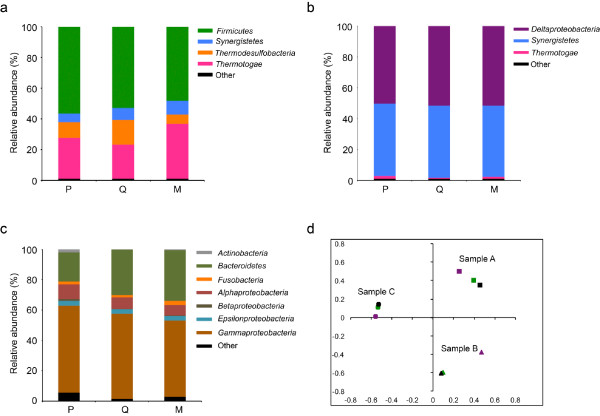
**Comparison of microbial communities based on 16S rRNA gene sequencing.** Relative abundance of bacterial phyla (class for *Proteobacteria*) from DNA extracted from **a**) sample A, **b**) sample B and **c**) sample C using the PowerBiofilm (P), QuickGene (Q) and Maxwell (M) extraction methods. Unclassified sequences and phyla (or class for *Proteobacteria*) with membership < 1% of total sequences were pooled into the classification labeled "Other". **d**) Non-metric multidimensional scaling (NMDS) plot based on *θ*_*YC*_ distances between libraries extracted using the PowerBiofilm (purple), QuickGene (green) and Maxwell (black) methods from Sample A (squares), B (triangles) and C (circles). AMOVA: p < 0.001.

To identify the major bacterial taxa present in the three samples, 454 pyrosequencing libraries of the V1-V2 region of 16S rRNA gene were generated (Figure 4). The number of sequences analyzed per 16S gene library were: 11907 (P), 12076 (Q), and 1050 (M) for sample A; 9754 (P) 8186 (Q) and 12634 (M) for sample B; and 11319 (P), 14705 (Q), and 13175 (M) for sample C. Although library sizes varied considerably, especially for sample A, the proportion of sequences that classified to the same taxonomic groups (at 97% similarity) was comparable among the DNA extracts (Figure 4). Furthermore, bacterial composition was very different between the three samples. For sample A, dominant phyla were gram-positive members of the *Firmicutes* (48-56%), and to a lesser extent *Thermotogae* (22-36%), *Thermodesulfobacteria* (6-16%) and *Synergistetes* (6-9%) (Figure 4a). The dominance of gram-positive *Firmicutes* in all three sample A extracts demonstrated that the three platforms were all capable of lysing these harder-to-lyse microorganisms. For sample B libraries, members of the phylum *Synergistetes* (46-47%) and the class *Deltaproteobacteria* (50-52%) were equally dominant among extracts, with minor representation by *Thermatogae* (0.7-2.0%) (Figure 4b). DNA extracts from the seawater-carrying pipeline sample C appeared more diverse than samples A and B, with dominant taxonomic groups that included members of the *Gammaproteobacteria* (49-56%), *Alphaproteobacteria* (7-10%), and *Bacteroidetes* (19-33%). Less abundant representation by *Epsilonproteobacteria* and *Fusobacteria* (2-3%) and the minor group of gram-positive *Actinobacteria* (0.1-1.8%) was also observed (Figure 4c). An AMOVA performed on a random subsample (1050 sequences) from each library demonstrated that samples clustered together regardless of extraction method and were significantly different from one another (p < 0.001) (Figure 4d). These data support the conclusions drawn from the DGGE analysis demonstrating: 1) microbial communities of the three samples differed from one another and 2) bacterial composition for a given sample was comparable among the three extraction methods. Furthermore, whether a dominant (sample A) or minor (sample C) group, gram-positive bacteria were detected by all three platforms with only minor variation between the three extraction methods.

**Figure 5:**
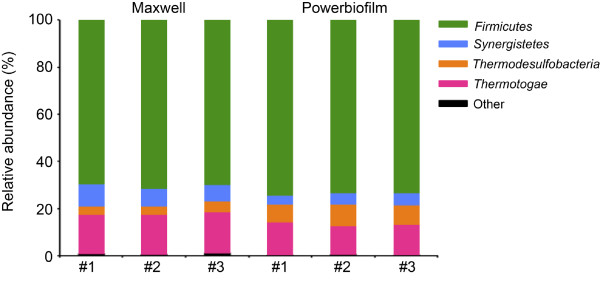
**Variation among sample A replicate libraries from PowerBiofilm and Maxwell extractions.** Relative abundance of bacterial phyla (class for *Proteobacteria*) from DNA extracted from sample A replicate libraries (n = 3 PowerBiofilm replicate libraries and n = 3 Maxwell replicate libraries). Unclassified sequences and phyla with membership < 1% of total sequences were pooled into the classification labeled "Other". AMOVA: p = 0.106.

Next, to rule out extraction bias as the sole source of variation observed between extraction platforms, technical replicates were compared (Figure 5, Additional file 1: Table S2). Sample A was chosen for this analysis, as a large proportion of its membership belongs to the gram-positive *Firmicutes* (Figure 4a). Three replicates of the Maxwell and PowerBiofilm extractions were compared, as they represent the most and least automated platforms, respectively (Table 1). The six sample A libraries (three Maxwell replicates and three PowerBiofilm replicates) contained a total of 14806 sequences that clustered into 308 OTUs at 97% similarity, 127 of which were singletons (i.e. OTU containing a single sequence). Analysis of a random number of sequences (n = 1958) from each library showed that although there was variation among all libraries (Additional file 1 Table S2) they were not significantly different from one another (AMOVA: p = 0.106). Furthermore, an analysis of the dominant phyla demonstrated that sequences from all replicates were classified as members of the same few genera: *Thermacetogenium, Halolactibacillus,* and *Thermoanaerobacter* for *Firmicutes* (phylum)*; Thermovirga* for *Synergistetes* (phylum); *Thermodesulfobacterium* for *Thermodesulfobacteria* (phylum); and *Kosmotoga, Thermotoga,* and *Thermosipho* for *Thermotogae* (phylum). Approximately 99% of the sequences were represented in both extraction methods. The ~1% of sequences exclusive to one or the other were either unclassified or sequences present in only one of the three replicates for a given extraction method.

## Discussion

In lieu of lengthy and potentially biased culturing methods, PCR-based analyses are an adequate and much less time-consuming alterative to monitoring microbial biofouling (Filion 2012). However, the interval between sample collection and analysis can influence the microbial community structure (Rochelle et al. 1994; van der Kraan et al. 2010) leading to erroneous results and complicating the ability to correctly assess fouling severity. Commercially available kits yield high-quality nucleic acids, but time-consuming sample processing and the requirements for additional equipment largely limits their use to molecular biology labs. Therefore, more automated nucleic acid extraction platforms were evaluated for potential use in performing DNA extractions in remote areas or with limited laboratory facilities. Automated platforms provide several practical advantages: 1) the ability to process samples in remote locations, 2) on-site extractions bypass the shipment of potentially hazardous samples, 3) reducing the training needed for personnel conducting the nucleic acid extraction, and 4) reducing time to implement corrective measures. Findings presented here demonstrated that the more automated methods were successful in extracting DNA from both high- and lower-biomass biofilm samples scraped from the inner surface of oil pipelines and that all three extraction platforms produced high-quality DNA suitable for PCR-based analyses.

The PowerBiofilm extraction platform included repeated vortexing, sample transfers, centrifugations, incubations, and additional equipment needed for processing steps. Personnel with some molecular biology experience are best suited for this level of sample manipulation. In addition to the need for technical expertise, variability in extraction reproducibility among subsamples (Figure 1a) warranted the consideration of alternate extraction platforms. The QuickGene platform also required manual steps for sample preparation and cell lysis, but the QuickGene-Mini80 instrument streamlined the binding, washing and elution using pressure filtration and could process up to eight samples simultaneously. Both QuickGene and PowerBiofilm platforms use column chromatography for DNA capture. QuickGene provided greater reproducibility and higher DNA yields for the high biomass samples, but still required ancillary equipment for sample preparation. The Maxwell method provided the best overall performance in terms of the ease of use and DNA yields for both high- and lower-biomass samples. The Maxwell 16 instrument, with the footprint of a microwave oven, is readily transportable for use in the field and can processes 16 samples simultaneously. Sample processing was completely automated, requiring no ancillary equipment and only minimal technical experience was required. These properties make the Maxwell system a better choice for DNA extractions at remote locations for the sample types tested.

Estimates of 16S rRNA gene copies per ml original sample corroborated the quantitative differences in DNA yields. Many variables between the three platforms could account for the differences in extraction efficiency. One variable that is correlated to efficiency is the format of the matrix used to bind DNA (Kephart et al. 2006). QuickGene utilizes a specialized high-capacity DNA-binding membrane ~1/12.5 the thickness of traditional glass membranes and the Maxwell uses silica-coated paramagnetic beads to bind nucleic acids. These beads are transferred to adjacent wells for washing and elution of DNA. The magnetic beads may have a greater binding-capacity or opportunity to bind DNA than the filter matrices used with the PowerBiofilm (or QuickGene) platform. The filter formats may also retain a greater amount of contaminating compounds, yet all downstream analyses indicated that the DNA from each method was of high quality. Differences in cell lysis between each extraction platform were identified as a potential concern, as differences in the community analysis may result if complete lysis was not achieved (Frostegård et al. 1999; Krsek and Wellington 1999). The PowerBiofilm and Maxwell platforms included physical disruption via bead-beating or plunging activity by a magnetic rod respectively, whereas lysis by the QuickGene platform was accomplished through sample rotation at elevated temperature. Both DGGE and pyrosequencing of PCR-amplified 16S rRNA genes, however, showed that the structures of the microbial communities surveyed were minimally affected by the method of DNA extraction (Figures 3 through 5). Importantly, the three extraction platforms showed similar proportions in the dominant gram-positive *Firmicutes* in sample A (56%-P, 53%-Q 48%-M), demonstrating that these three extraction platforms were capable of lysing cells with tough cell walls, which may be present in other complex samples.

The conclusion drawn from pyrosequencing data was made with caution as variation between technical replicates, replicate samples, and identical samples from one sequencing run to another has been documented (Zhou et al. 2011 and Schloss et al. 2011). With the number of singletons (single sequence-containing OTUs) ranging from 12 to 33 for each sample A replicate library, the variation observed for the OTU analysis was expected, as was that observed between the separate sequencing runs for the single (Figure 4) versus replicate (Figure 5) sample A analyses (i.e. gram-positive *Firmicutes* remained dominant at 50-60% and 70-75%, respectively). Therefore, while biases between extraction methods are noted in the literature (Stach et al. 2001), the variation observed in 454 pyrosequencing studies presented here may be primarily the result of variation arising during post-nucleic acid extraction processes (Schloss et al. 2011; Zhou et al. 2011).

The Promega Maxwell 16 platform's portability and ease of use make it an attractive alternative to manual extractions if space is a limitation. The Maxwell 16 has several advantages over the Powerbiofilm and QuickGene Mini-80 platforms. First, the Maxwell 16 requires no additional equipment for sample processing, resulting in minimal sample handling. Second, the small size allows transport for use in mobile labs, where samples taken from remote sites could be processed within hours of procurement. Third, up to 16 samples are ready for PCR-based analyses within an hour of processing ensuring that shifts in bacterial communities are minimal. We conclude that the QuickGene and Maxwell platforms are examples of suitable alternatives for molecular analysis of microbial biofouling, and that automated DNA extraction platforms from a variety of manufacturers may facilitate microbial contaminant assessment in many industrial settings.

## Competing interests

The authors declare that they have no competing interests. None of the authors are employed by or have any financial stake in any of the companies represented in this manuscript aside from the loan of equipment for demonstration comparisons.

## Supplementary Material

Additional file 1 **Table S1.doc** List of 8nt barcodes for 454 pyrosequencing. Barcodes for each 454 pyrosequencing library.**Table S2.doc** Measures of alpha diversity for each PowerBiofilm and Maxwell replicate library. Alpha diversity matrices for sample A replicate libraries. Click here for file
