# Supplementary material for: Automated DNA extraction platforms offer solutions to challenges of assessing microbial biofouling in oil production facilities
Source: AMB Express. 2012 Nov 20;2:60. doi: 10.1186/2191-0855-2-60 (PMC3539857; doi:10.1186/2191-0855-2-60)
Supplement: Additional file 1 — Table S1.doc List of 8nt barcodes for 454 pyrosequencing. Barcodes for each 454 pyrosequencing library.Table S2.doc Measures of alpha diversity for each PowerBiofilm and Maxwell replicate library. Alpha diversity matrices for sample A replicate libraries. [file 2191-0855-2-60-S1.pdf]

Article title: Automated DNA Extraction Platforms Offer Solutions to Challenges of Assessing Microbial Biofouling in Oil Production Facilities

Journal name: AMB Express

Author names: Athenia L. Oldham, Heather S. Drilling, Blake W. Stamps, Bradley S. Stevenson, Kathleen E. Duncan

Corresponding author: Athenia L. Oldham, University of Oklahoma, 770 Van Vleet Oval GLCH 136, Norman, OK 73019, USA. email: athenia.L.oldham-1@ou.edu; phone: 405-625-0535; fax: 405-325-3180

Table S1

List of 8nt barcodes for 454 pyrosequencing.

**Barcode sequence**	**Sample**	**Extraction Library**
CACAACTG	A	Maxwell
CACAACAC	A	PowerBiofilm
CAAGTGGA	A	QuickGene
CACACACA	B	Maxwell
CACAAGTC	B	PowerBiofilm
CACAAGAG	B	QuickGene
CACACTGA	C	Maxwell
CACACTCT	C	PowerBiofilm
CACACAGT	C	QuickGene
CAAGGAAC	A	Maxwell replicate #1
CAAGGATG	A	Maxwell replicate #2
CAAGGTAG	A	Maxwell replicate #3
CAAGGTTC	A	PowerBiofilm replicate #1
CAAGTCCA	A	PowerBiofilm replicate #2
CAAGTCGT	A	PowerBiofilm replicate #3

Table S2

Measures of alpha diversity for each library.[a]

Sample A library	% Library coverage[b]	OTUs[c]	Chao[d]	ACE[e]	H^[f]	Simpson (D)[g]
Maxwell#1	0.964	122	308 (215,495)	455 (371,567)	2.76	0.157 (0.145,0.168)
Maxwell#2	0.972	102	191 (145,286)	305 (248,384)	2.20	0.310 (0.289, 0.331)
Maxwell#3	0.976	107	150 (128,197)	218 (182,273)	2.28	0.297 (0.276, 0.317)
Powerbiofilm#1	0.972	103	187 (144,275)	198 (153,282)	2.67	0.162 (0.150, 0.175)
Powerbiofilm#2	0.966	118	276 (197,434)	387 (316,482)	2.83	0.132 (0.123, 0.142)
Powerbiofilm#3	0.967	114	253 (184,390)	361 (289,463)	2.69	0.174 (0.161, 0.188)

[a]Based on a random subsampling of 1958 sequences.
[b]Good's coverage for an OTU definition.
[c]OTU: operational taxonomic units
[d]Chao richness estimator, mean value (95% low and high confidence intervals).
[e]Abundance based coverage estimator (ACE), mean value (95% low and high confidence intervals).
[f]Non-parametric estimate of the classical Shannon diversity index (H^).
[g]Simpson index, mean value (95% low and high confidence intervals).
